# Terahertz Faraday Rotation of SrFe_12_O_19_ Hexaferrites Enhanced by Nb Doping

**DOI:** 10.1021/acsami.2c13088

**Published:** 2022-10-04

**Authors:** Zimeng Hu, Gavin B. G. Stenning, Vladimir Koval, Jiyue Wu, Bin Yang, Alisa Leavesley, Richard Wylde, Michael John Reece, Chenglong Jia, Haixue Yan

**Affiliations:** † School of Engineering and Materials Science, Queen Mary University of London, Mile End Road, LondonE1 4NS, United Kingdom; ‡ ISIS Neutron and Muon Source, Rutherford Appleton Laboratory, Didcot, OxfordshireOX11 0QX, United Kingdom; § Institute of Materials Research, Slovak Academy of Sciences, Kosice04001, Slovakia; ∥ Faculty of Science and Engineering, University of Chester, Parkgate Road, ChesterCH1 4BJ, United Kingdom; ⊥ Virginia Diodes Inc., 979 2nd St SE #309, Charlottesville, Virginia22902, United States; # Thomas Keating Ltd, Billingshurst, West SussexRH14 9SH, United Kingdom; ∇ Key Laboratory for Magnetism and Magnetic Materials of MOE, Lanzhou University, Lanzhou730000, P. R. China

**Keywords:** SrFe_12_O_19_ hexaferrite, THz, Faraday rotation, ferrimagnetic, dielectric

## Abstract

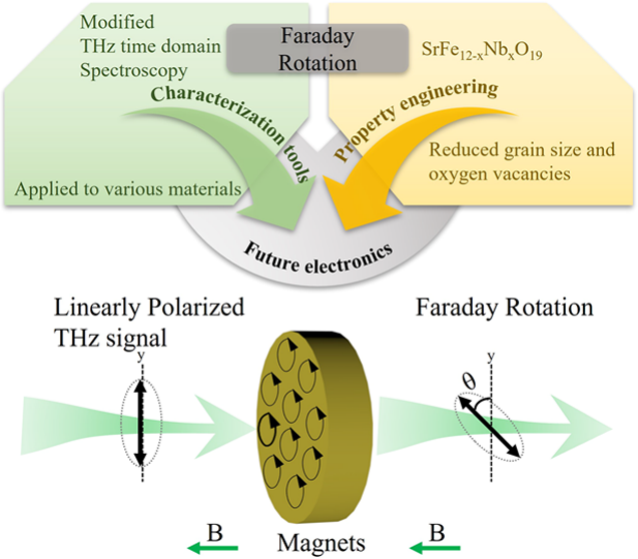

The magneto-optical and dielectric behavior of M-type hexaferrites as permanent magnets in the THz band is essential for potential applications like microwave absorbers and antennas, while are rarely reported in recent years. In this work, single-phase SrFe_12–*x*_Nb_*x*_O_19_ hexaferrite ceramics were prepared by the conventional solid-state sintering method. Temperature dependence of dielectric parameters was investigated here to determine the relationship between dielectric response and magnetic phase transition. The saturated magnetization increases by nearly 12%, while the coercive field decreases by 30% in the *x* = 0.03 composition compared to that of the *x* = 0.00 sample. Besides, the Nb substitution improves the magneto-optical behavior in the THz band by comparing the Faraday rotation parameter from 0.75 (*x* = 0.00) to 1.30 (*x* = 0.03). The changes in the magnetic properties are explained by a composition-driven increase of the net magnetic moment and enhanced ferromagnetic exchange coupling. The substitution of the donor dopant Nb on the Fe site is a feasible way to obtain multifunctional M-type hexaferrites as preferred candidates for permanent magnets, sensors, and other electronic devices.

## Introduction

Magnetic materials like hexaferrites^1,2^ and spinel ferrites^3,4^ have been investigated for decades due to their advanced electronic and magnetic performances and potential applications. M-type hexaferrites having the general formula MeFe_12_O_19_ (Me is a divalent ion like Ca, Sr, Ba, Pb, etc.) are widely studied for sensing and imaging applications as well as for advanced multi-state memory devices, transducers, and RF/MW filters.^5,6^ Their unique magnetic, dielectric, and multiferroic properties originate from their large magneto-crystalline anisotropy along the *c*-axis and collective displacement of iron ions in the FeO_5_ bipyramidal units. The crystal structure of M-type hexaferrites is hexagonal with the space group *P*6_3_/*mmc*. The *P*6_3_/*mmc* unit cell consists of RSR*S* layers, where S = Fe_6_O_8_^2+^ is the spinel block and R = MeFe_6_O_11_^2–^ is the hexagonal block. The R*S* layers are RS layers rotated around the *c*-axis by 180°. Fe^3+^ ions occupy five different sites showing opposite spin rotations: at the 12k, 2b, and 2a octahedral sites, the spins have the up ↑ direction, and at the 4f_1_ and 4f_2_ sites, the spins are aligned in the down ↓ direction.^7^ As a consequence, the M-type hexaferrites show ferrimagnetic behavior at room temperature.

Among the M-type hexaferrites, SrFe_12_O_19_ has become one of the most studied hard ferrites due to its high coercive field (*H*_c_ = 5.55 kOe),^8^ high Curie temperature (*T*_c_ = 460 °C),^9^ large saturation magnetization (*M*_s_ = 0.056 emu/mg), and large remnant magnetization (*M*_r_ = 0.016 emu/mg).^2,9^ SrFe_12_O_19_ can be prepared at a low cost, which makes it an attractive material for commercial use. However, achieving larger saturated magnetization and, at the same time, appealing dielectric behavior remains a big challenge in designing and preparing high-performance hexaferrites derived from SrFe_12_O_19_.

The most promising approach to synthesize SrFe_12_O_19_-based hexaferrites and tailor their functional properties for desired applications is a partial substitution of Sr^2+^ ions by isovalent Ba^2+^, Pb^2+^, and Ca^2+^ ions^10−13^ or trivalent rare earth elements^14^ like La^3+^, Nd^3+^, and Sm^3+^. Doping at Sr sites by the La^3+^ ion with smaller radii has been reported to decrease both saturated and remnant magnetization of SrFe_12_O_19._^14,15^ It was shown that the La^3+^ substitution makes the valence variation from Fe^3+^ to Fe^2+^ and the noncollinear spin arrangement of magnetic moments, which results in this decrement of magnetization.^14^ In addition, substitution with rare earth elements can significantly increase the grain size of SrFe_12_O_19_-based ceramics, further enlarging the magnetic coercive field.^14^

Another strategy to improve the functional properties of the M-type hexaferrites is a partial substitution of the Fe ions by dopants: (i) isovalent ions such as Sc^3+^,^16^ Ga^3+^,^17^ Al^3+^,^18,19^ and In^3+^;^20^ (ii) Co^2+^ ions^21,22^ or Nb^3+^;^23^ and (iii) ionic combinations like Cr^3+^–Zn^2+^,^24^ Co^4+^–Ca^2+^,^21^ or Zr^4+^–Cd^2+^.^7^ For example, the coexistence of electrical and magnetic ordering at room temperature was observed in modified SrFe_12–*x*_In_*x*_O_19_ magnetoelectric multiferroic ceramics.^25,26^ The cointroduction of Co^4+^ and Ca^2+^ ions on Fe sites in BaFe_12_O_19_ can increase the dielectric permittivity if compared to the undoped one due to a higher concentration of Fe^3+^ ions in the high spin state.^21^ The reduced grain size, increased saturated magnetization, and large magneto-crystalline anisotropy were obtained in the Nb-substituted BaFe_12_O_19._^23^ In addition, the introduction of Nb^3+^ ions can help decrease both the alternating current (AC) conductivity and direct current (DC) conductivity of the M-type hexaferrites, which suggests the potential function of Nb for improving the dielectric behavior.^27^ Asghar and Anis-ur-Rehman have proposed, based on the Maxwell–Wagner two-layer theory, that the highly resistive grain boundaries are responsible for the reduced conductivity of hexaferrites in the dielectric measurements.^24,28^ The above-mentioned AC studies, however, were carried out on M-type hexaferrites within a narrow frequency range. To date, there have been only a few reports on the dielectric behavior of M-type hexaferrites at terahertz (THz) frequencies^29,30^ and much less study on the Faraday rotation,^31^ knowledge of which is crucial for the construction of optical communication devices. Moreover, a comprehensive study on the dielectric properties of hexaferrites over a wide frequency and temperature range is missing. Also, for future perspectives, it is also necessary to search for new hexaferrites with tunable dielectric, magnetic, and even magnetodielectric properties.

As the Nb^5+^ ion can electrically compensate for the presence of the Fe^2+^ ions and simultaneously inhibit the abnormal grain growth in polycrystalline SrFe_12_O_19_, a study on the dielectric and magnetic properties of the SrFe_12_O_19_ ceramics (with and without Nb doping) was undertaken to explore the relationship between the composition, structure, and functional properties of these hard ferrites. Here, two compositions of SrFe_12–*x*_Nb_*x*_O_19_ (*x* = 0.00 and 0.03) were designed. Additionally, for the first time, a modified technique of THz spectroscopy is introduced to study the Faraday rotation effect in hexaferrites. Finally, using this technique, we demonstrate that the Nb-doped SrFe_12_O_19_ ceramics possess a large relative permittivity and Faraday rotation at THz frequencies, suggesting that the Nb-modified M-type hexaferrites are useful in optical communication devices, security surveillance systems, and sensing applications.

## Materials and Methods

### Materials

Hexaferrite ceramics can be prepared by the solid-state sintering method,^22^ sol–gel method, and green pulsed laser ablation in liquid (PLAL) approaches.^32,33^ Here, SrFe_12–*x*_Nb_*x*_O_19_ ceramics, with *x* = 0.00 and 0.03 (abbreviated as SFO and SFN3O), were prepared by the conventional solid-state method using raw materials of SrCO_3_ (purity ≥ 99.9%, Aldrich), Nb_2_O_5_ (purity ≥ 99.9%, Alfa Aesar)_,_ and Fe_2_O_3_ (purity ≥ 99.945%, Alfa Aesar). The chemicals were preheated at 200 °C for 24 h and then weighed according to the stoichiometric formula. They were ball milled in ethanol for 12 h at 250 rpm using stainless balls and vessels. The slurry was dried, and the powder product was calcined at 1100 °C for 6 h. To reduce the particle size, the calcined powder was ball milled again. The fine precursor was mixed with 5 wt % PVA and then pressed into pellets with a diameter of 13 mm and thickness of 1–2 mm. The pellets were heated at 800 °C for 2 h in air to remove the binder. Sintering was carried out at 1200 °C for 6 h in air. The sintered pellets were polished and then annealed in air for 12 h at 1000 °C.

### Methods

The crystal structure of the sintered ceramics was investigated by X-ray powder diffraction (XRD, PANalytical, Cubix) on crushed powders using Ni-filtered Cu Kα radiation (λ = 1.5418 Å) over the 2θ range of 5–120° with a step of 0.0315°. Structural analysis was performed using Rietveld refinement using the EXPGUI and GSAS software packages.^34,35^ The surface morphology of the polished and thermally etched samples was observed by scanning electron microscopy (SEM). Surface element analysis was performed with an X-ray photoelectron spectrometer (XPS, Nexsa). Thermal analysis was carried out by differential scanning calorimetry (DSC, rheometric scientific, a model STA 1500 H) in N_2_ from 25 to 800 °C with a heating/cooling rate of 10 °C/min. For dielectric measurements, the as-sintered samples were ground to less than 0.5 mm thickness and then coated with silver paint (Gwent Electronic Materials Ltd., C2011004D5, Pontypool, U.K.). The temperature dependence of the relative dielectric permittivity (ε*′*) and loss tangent (tan δ) were measured in the temperature range 25–600 °C at three different frequencies (100 kHz, 500 kHz, and 1 MHz) via a computer-controlled system with an LCR meter (Agilent, 4284A) attached to a furnace. The field-dependent magnetization (*M*–*H*) loops of the samples at room temperature and the zero-field cooling (ZFC) and field cooling (FC) magnetizations were measured over the temperature range 1.8–400 K at 1000 Oe using a superconducting quantum interference device (SQUID, Quantum Design). The dielectric properties and Faraday rotation in the THz region were measured by modified terahertz time-domain spectroscopy (THz-TDS, TeTechs Ltd., Canada) in transmission mode. Electromagnetic radiation ranging from 0.2 to 0.8 THz was used to illuminate tiny wafers with a thickness of 1 mm and a diameter of 12 mm. The collected THz time-domain spectra were Fourier transformed to obtain both amplitude and phase information in the frequency domain. All information in the frequency domain was used to extract the permittivity and loss tangent data of the samples.^31,36^ The permittivity and loss tangent in the THz band were converted from the refractive index of virgin samples. After magnetizing at a magnetic field of 3500 Oe, the samples were tested in the THz band with right-handed and left-handed gratings to study the Faraday rotation effect.

## Results and Discussion

**Figure 1:**
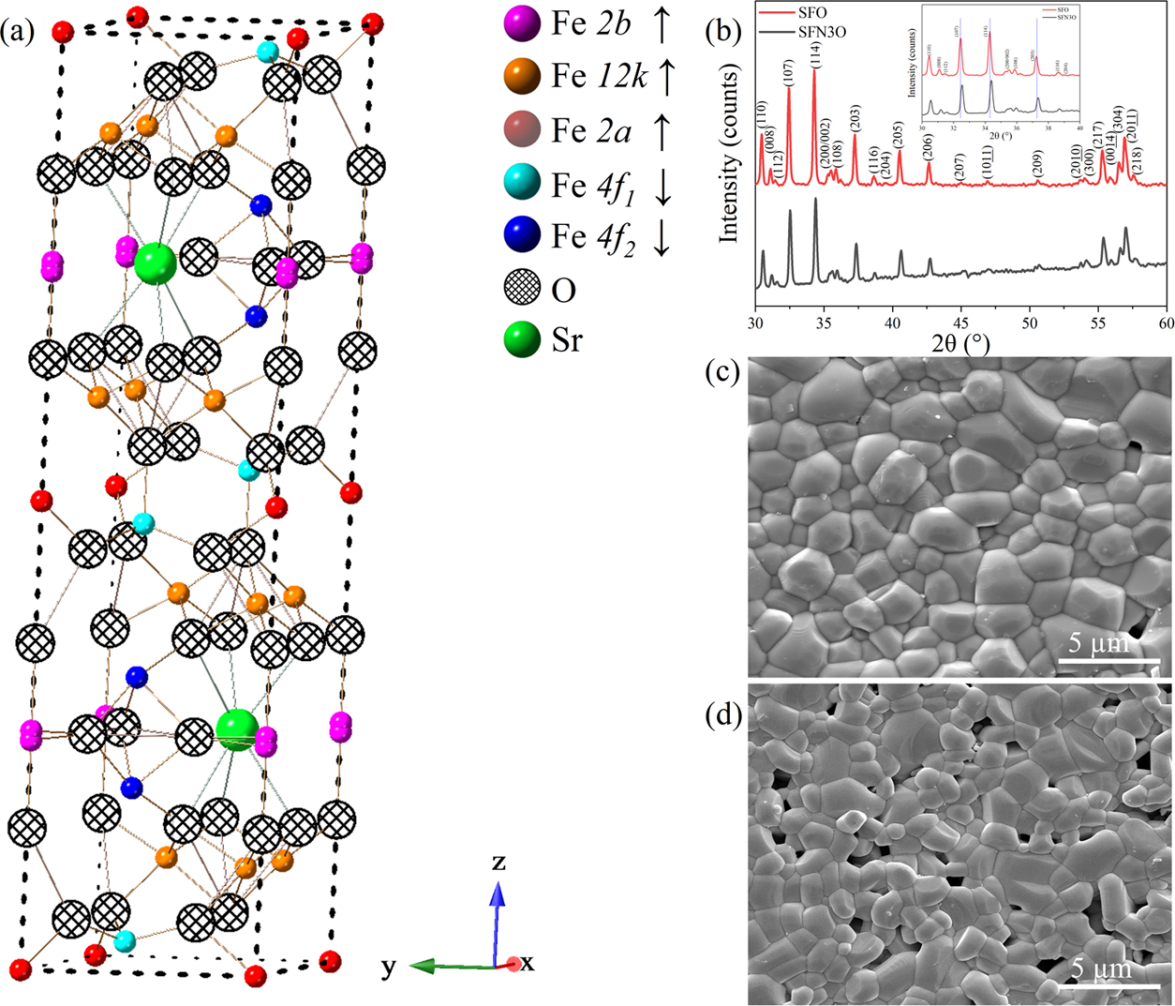
(a) Schematic of the crystal structure of pure SrFe_12_O_19_ hexaferrite. (b) XRD patterns of SrFe_12–*x*_Nb_*x*_O_19_ (*x* = 0.00 and 0.03) ceramics within the selected 30–60° range; indexing was performed based on the reference standard SrFe_12_O_19_ (ICSD no. 69022). Inset: enlarged view of XRD patterns within 30–40°. (c, d) SEM images of the thermally etched ceramics with *x* = 0.00 and 0.03 ceramics, respectively.

A schematic of the crystal structure of SrFe_12_O_19_ hexaferrite is illustrated in Figure 1a. Figure 1b shows the room-temperature XRD patterns of the SrFe_12–*x*_Nb_*x*_O_19_ (*x* = 0.00 and 0.03) ceramics. Both SFO and SFN3O are single-phase materials with a hexagonal structure (space group: *P*6_3_/*mmc*). The Miller indices are labeled based on the reference SrFe_12_O_19_ standard (ICSD no. 69022) and are in good agreement with Kimura’s work on the structural analysis of SrFe_12_O_19._^18^ The Nb^5+^ ions can enter both the octahedral and tetrahedral sites based on previous work.^23,37^ Here, Nb^5+^ ions prefer occupying the spin-down 4f_1_ and 4f_2_ sites. The well-fitted XRD patterns within the selected range of 20–120° for SFO and SFN3O are shown in Figure S1a,b, respectively. The χ^2^ factor for good fitting does not exceed 2.7. The refinement and crystal parameters are listed in Table S1. In diffractograms, no secondary phase is observed within the detection limit of the X-ray diffractometer. Thus, the substitutional niobium ions are supposed to incorporate into the lattice. Because of the smaller ionic radius of Nb^5+^ (0.640 Å) compared to that of Fe^3+^ (0.645 Å),^38^ the volume of the unit cell decreases on doping from 692.887(2) to 692.262(3) (Å^3^), and thus the shifting of diffraction peaks toward higher angles is observed for SFN3O in Figure 1b (inset), indicated by blue short-dotted lines. The etched microstructures of the SFO and SFN3O ceramics are shown in Figure 1c,d. As can be seen, the grains are closely packed in both samples. This observation is consistent with the high relative density of the ceramics (>95%) measured using the Archimedes’ method. It should be noticed that the grain size decreases with Nb doping from 1.85 μm (SFO) to 1.43 μm (SFN3O), which is in accordance with SEM observations of other Nb-doped dielectric ceramics.^39^ It should be noted that decreasing grain size results in a smaller unit cell volume due to larger surface tension forces, as reported in other magnets.^40,41^

**Figure 2:**
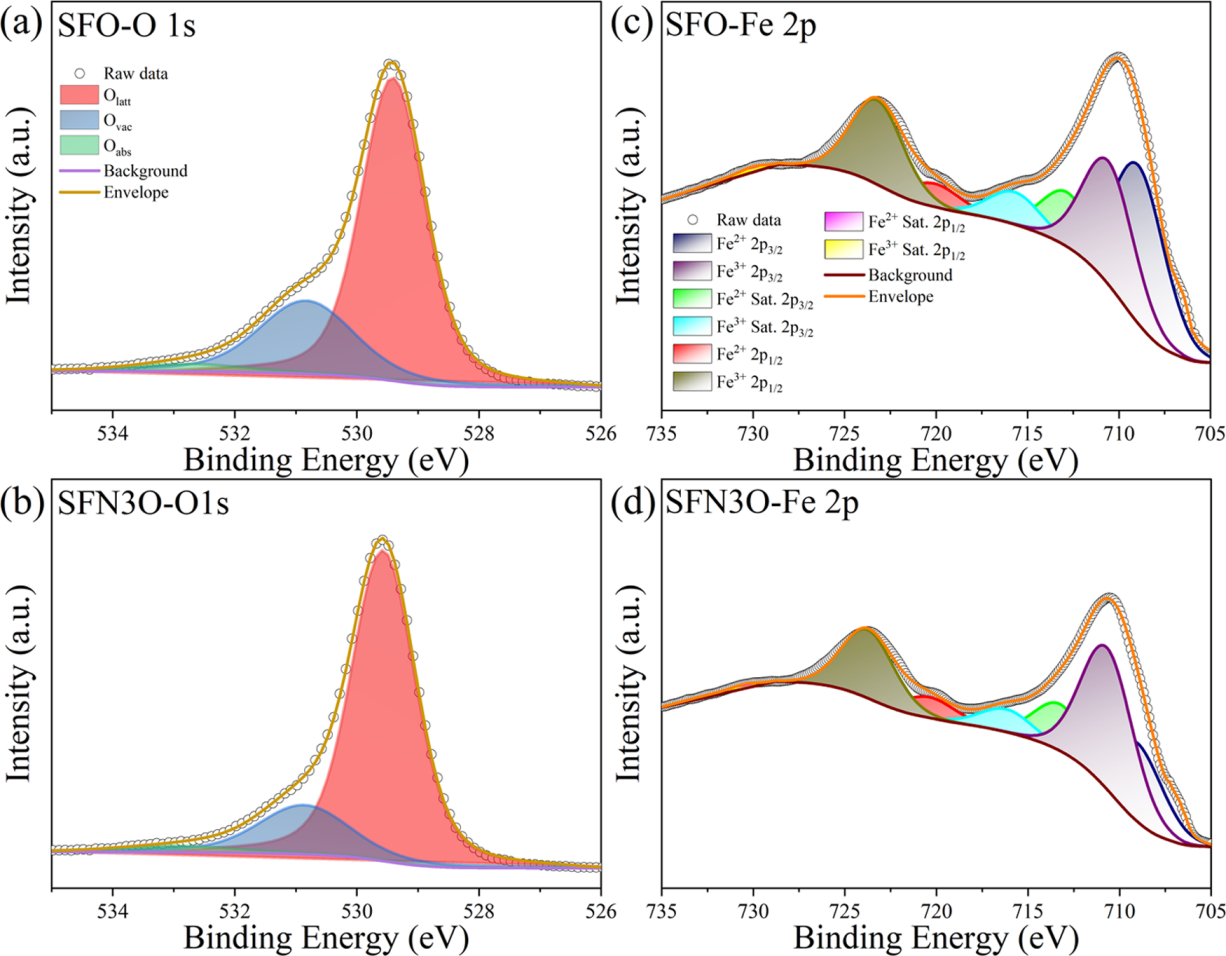
(a, b) Fitted O 1s XPS spectra and (c, d) fitted Fe 2p XPS spectra for the SFO and SFN3O hexaferrites, respectively.

The dielectric and magnetic properties of hexaferrites strongly depend on the content of Fe ions and oxygen vacancies.^29^ Therefore, information on the oxidation state of Fe, which is closely related to oxygen vacancies and changes due to processing at high temperatures, is of great importance. To explore the effect of the Nb substitution on the valence of Fe in the prepared hexaferrites, X-ray photoelectron spectroscopy was employed. The fitted O 1s XPS spectra of SFO and SFN3O are shown in Figure 2a,b. The spectra were fitted by the Avantage software using the Gaussian–Lorentzian product (GLP).^42^ The results of fittings are summarized in Table S2. Apparently, the experimental O 1s spectrum is formed by three spectral peaks–the red curve peak corresponds to the lattice oxygen (*O*_latt_), the blue peak represents oxygen in a deficient environment (*O*_vac_), and the green curve peak can be ascribed to chemisorbed or dissociated oxygen (*O*_abs_) from the air.^43,44^ The ratio of integrated areas of *O*_vac_:*O*_latt_ can be used to compare the change in the relative oxygen vacancy concentration between compositions.^45^ The binding energies of the *O*_latt_, *O*_vac_, and *O*_abs_ peaks in SFO are 529.40, 530.82, and 532.78 eV, respectively. In SFN3O, the respective peaks are shifted to 529.56, 530.86, and 532.99 eV. The reduced ratio *O*_vac_:*O*_latt_ varies from 0.354(3) (SFO) to 0.222(1) (SFN3O), as calculated from the integral area of the corresponding peaks. Therefore, one can conclude that the concentration of oxygen vacancies decreases with Nb doping.^46^ The reduced oxygen vacancies are expected to improve the dielectric properties of the Nb-doped hexaferrites.^29^

To further investigate the origin of oxygen vacancies, the valence of Fe ions was analyzed by fitting the Fe 2p spectra, as shown in Figure 2c,d. The fitted results are listed in Table S3. The Fe 2p spectrum is formed by a typical doublet of Fe 2p_3/2_ and Fe 2p_1/2_ with satellite peaks corresponding to different valences of Fe.^29,47,48^ Deconvolution of the characteristic Fe 2p_3/2_ photon emission peak in SFO yields a doublet with the Fe^3+^ 2p_3/2_ (∼710.88 eV) and Fe^2+^ 2p_3/2_ (∼709.18 eV) peaks, and in SFN3O, the doublet is composed of the Fe^3+^ 2p_3/2_ (∼711.08 eV) and Fe^2+^ 2p_3/2_ (∼709.38 eV) peaks (depicted by the purple and blue curves in Figure 2c,d). From the fitted spectra, a fraction of the Fe ions in the two chemical states was determined by the integrated area ratio. It was found that the area ratio of Fe^2+^ 2p_3/2_: Fe^3+^ 2p_3/2_ decreases from 1.076(8) in SFO to 0.592(3) in SFN3O, which indicates that the reduction of Fe^3+^ is suppressed by the Nb^5+^ doping. The increased oxidation degree of Fe ions reflected by XPS data is consistent with decreased oxygen deficiency discussed before, which also agrees well with the previous work.^49^ In addition, the decreased oxygen deficiencies agree well with the smaller unit cell volume for SFO.

A phase evolution analysis of the SFO and SFN3O hexaferrites was performed using the DSC thermograms, as recorded on heating and cooling (Figure S2). Three thermal events denoted as *a*, *b*, and *c* are observed on the DSC curves for both samples. The first event *a* occurring at around 100 °C (on heating) indicates volatilization of the absorbed water.^50^ The other two events *b* and *c* can be linked with magnetic phase transitions. For SFO, the thermal feature *b* is around 290 °C and *c* is around 465 °C; the latter agrees well with the reported ferromagnetic to paramagnetic phase transition temperature (Curie temperature, *T*_c_) of pure SrFe_12_O_19_ hexaferrite, 470 °C.^9,51^ The underlying mechanism of the *b* event needs further investigation. Similar thermal events were observed for SFN3O; event *b* occurred at about 260 °C, and *c* occurred at around 490 °C.

**Figure 3:**
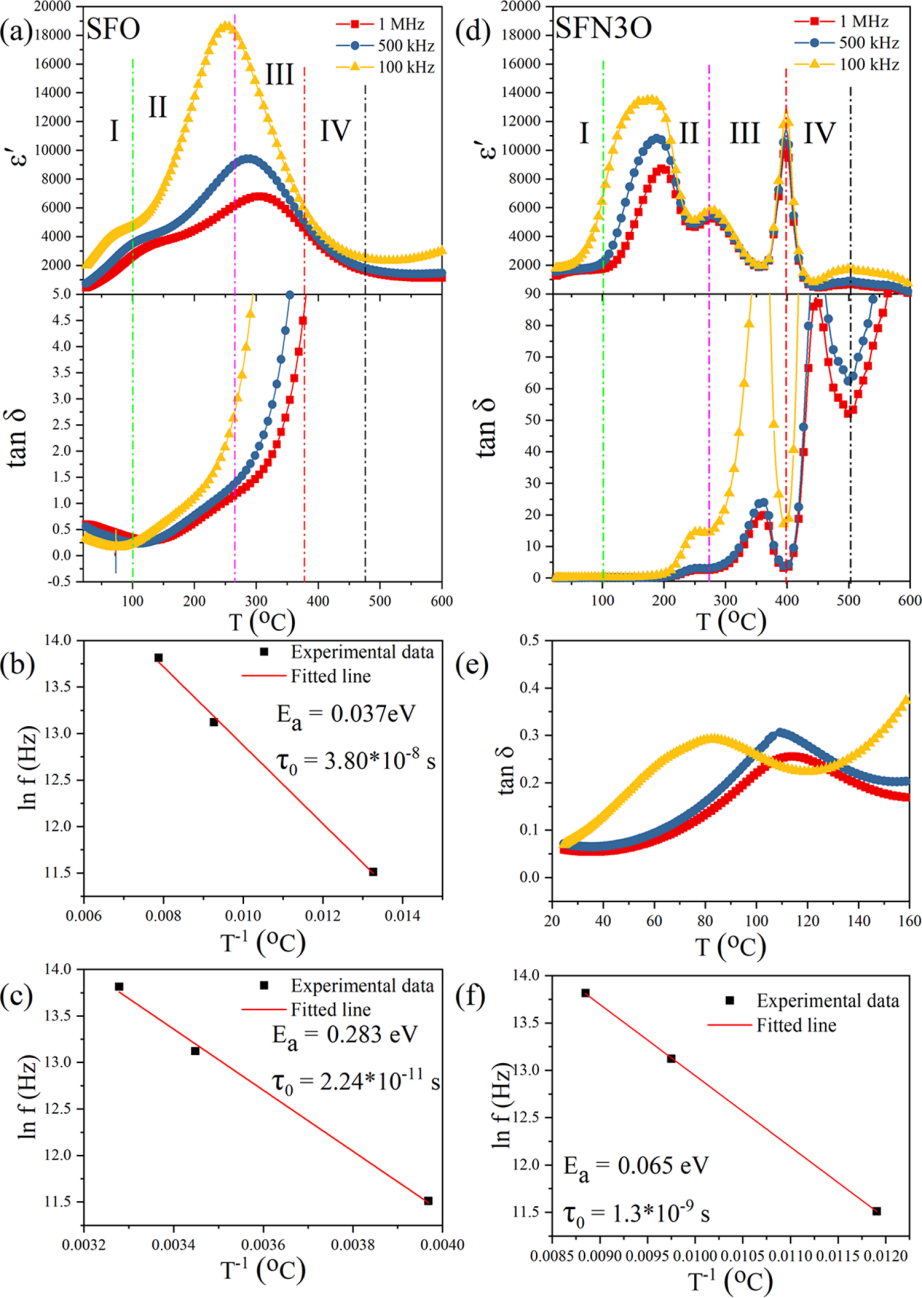
Temperature dependencies of the relative dielectric permittivity (ε*′*) and loss tangent (tan δ) of SFO (a) and SFN3O (d). (b, c) Arrhenius law fittings of the dielectric peaks of SFO in regions I and II, respectively. (e) Enlarged view of the loss tangent of SFN3O with a strong frequency dependence at temperatures between 25 and 160 °C. (f) Arrhenius law fitting of the dielectric peak in region II for the SFN3O hexaferrite.

To further study the phase transition behavior, the temperature dependencies of the relative dielectric permittivity (ε′) and loss tangent (tan δ) of the SFO and SFN3O ceramics were measured in the temperature range of 25–600 °C at three different frequencies, namely 100 kHz, 500 kHz, and 1 MHz (Figure 3a,d). At the Curie temperature, the arrangement of spins in ferrites changes from the long-range ordered ferromagnetic state to a paramagnetic state with random orientations. The magnetic transitions are usually accompanied not only by changes in the magnetic properties but also by variations in other physical properties, such as dielectric permittivity, specific heat, and so on.^52^ For SFO, two broad dielectric anomalies with a strong frequency dependence are observed in ε*′*(*T*) in regions I and II (Figure 3a). It should be noted that the dielectric permittivity reflects the ability of electric dipoles to oscillate in an applied AC field.^53^ Hence, the large decrease of the permittivity with increasing frequency from 100 kHz to 1 MHz can be explained by a lower contribution of interfacial polarization or point defects to the permittivity at higher frequencies. The second dielectric anomaly over regions II and III corresponds to the thermal feature *b* in Figure S2 and indicates a magnetic phase transition. The maximum of the permittivity occurs at a characteristic temperature, which shifts toward higher temperatures with increasing frequency. The frequency dispersion and diffusion of both dielectric peaks (in region I and through regions II and III) suggest that relaxation in the SFO hexaferrite can be attributed to point defects. The relaxation behavior should obey the Arrhenius law,^54,55^ as shown in Figure 3b,c.
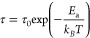
1

where τ is the relaxation time of defects, τ_0_ is a time constant, *E*_a_ is the activation energy, *k*_*B*_ is the Boltzmann constant, and *T* is the temperature linked with the maximum of ε′. The fitted activation energy *E*_a_ (0.037 eV) and relaxation time τ_0_ (3.8 × 10^–8^ s) for the first anomaly of SFO at around 100 °C suggest that the dielectric peak in region I is due to point defects, such as oxygen vacancies with long relaxation time. The second dielectric anomaly in the overlapping regions II and III is characterized by the values of *E*_a_ = 0.283 eV and τ_0_ = 2.24 × 10^–11^ s, which are characteristic of oxygen vacancies.

For the SFN3O sample, four dielectric anomalies with different dependence on frequency are observed at temperatures between 25 and 600 °C. In contrast to SFO, there is no anomaly in region I. With increasing temperature above 100 °C, a nearly frequency-independent permittivity anomaly occurs at around 270 °C in region II (Figure 3d). The corresponding loss peak is presented in Figure 3e. The fitted relaxation time τ_0_ = 1.30 × 10^–9^ s (see Figure 3f for the Arrhenius law fitting) is similar to that obtained for SFO, suggesting a contribution to the dielectric permittivity from defects. Instead, the dielectric anomaly of the SFN3O ceramic at 270 °C can be linked with a magnetic phase transition, which is consistent with the results of the DSC analysis (the event *b* in Figure S2b). The third, most intense anomaly is a frequency-independent feature occurring at about 400 °C. It is assumed that the loss peak at a slightly lower temperature (∼370 °C) is caused by enhanced domain wall activity, typical of ferroelectric materials.^53,56^ It should be mentioned that the fourth dielectric anomaly at around 500 °C shows a diffuse behavior but without a temperature shift. Moreover, this temperature is close to the thermal event *c* in the DSC curve (Figure S2b), suggesting the ferrimagnetic-to-paramagnetic phase transition. It is obvious that the Nb substitution increases the Curie temperature of the SrFe_12_O_19_ hexaferrite, which agrees well with the earlier study of Wang et al.^57^ This finding is further supported by the loss tangent minimum observed close to 500 °C.^56^ The origin of the dielectric anomalies (either the phase transition or point defects) is still under debate. Further studies are necessary to clarify the anomalous high-temperature critical behavior of hexaferrites.

**Figure 4:**
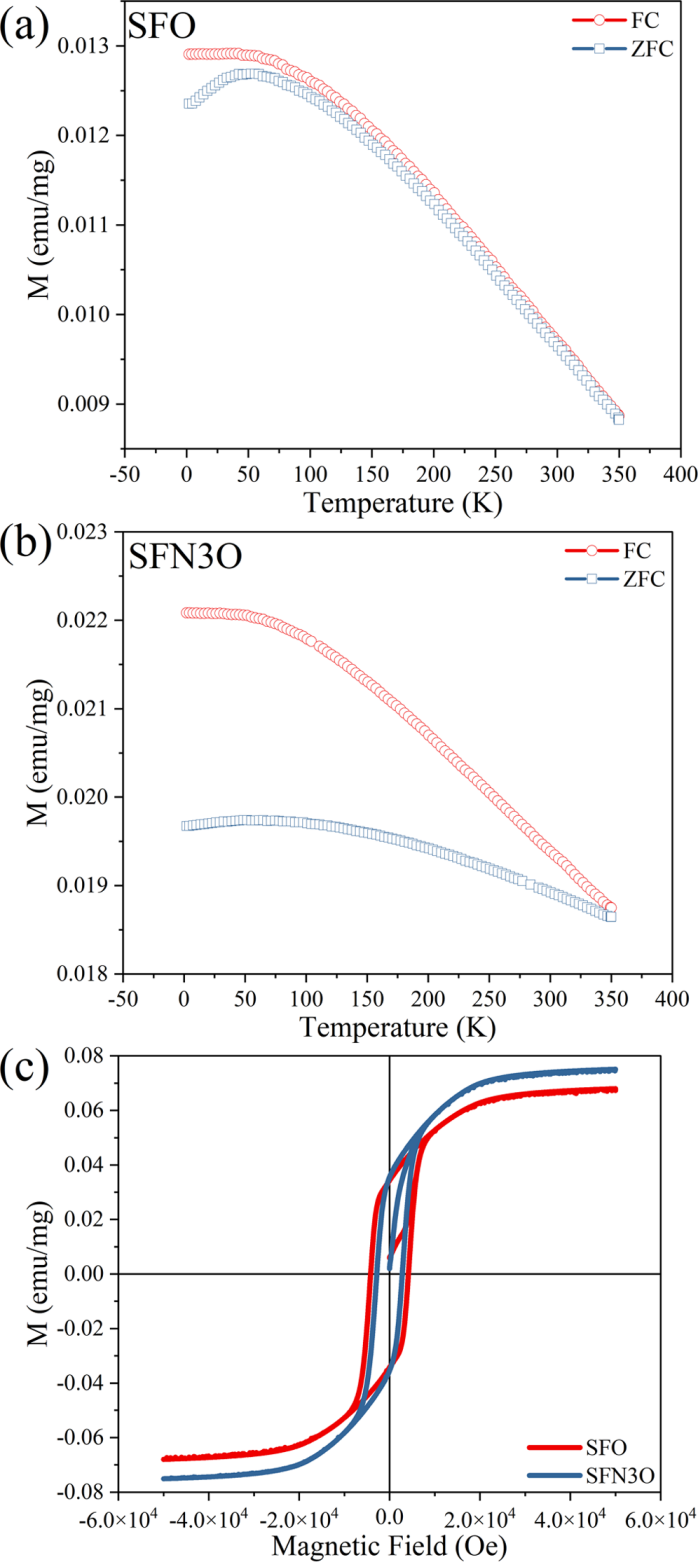
(a, b) ZFC and FC magnetization curves for SFO and SFN3O, respectively. (c) *M–H* hysteresis loops for SFO and SFN3O measured at 300 K.

The field cooling (FC) magnetization and zero-field cooling (ZFC) magnetization as a function of temperature for the respective SFO and SFN3O samples are shown in Figure 4a,b. At cryogenic temperatures, the FC magnetization increases from 0.013 emu/mg for SFO to 0.022 emu/mg for SFN3O. This increment of magnetization corresponds to the higher saturated magnetization *M*_s_ of SFN3O (see Table 1), as obtained from the *M*–*H* hysteresis loops in Figure 4c. For SFO and SFN3O, both the ZFC and FC magnetizations increase monotonously upon cooling from 300 K down to 100 K. Below 100 K, a plateau-like hump is observed due to the super spin-glass (SSG) behavior.^58^ Humbe et al.^59^ have reported on the magnetization peak in hexaferrites occurring at the blocking temperature (*T*_b_), where a magnetic structure changes from a superparamagnetic to ferrimagnetic one. No other peak corresponding to a possible phase transition from ferrimagnetic to paramagnetic phase is observed in Figure 4a,b for the respective SFO and SFN3O ceramics over a temperature range of 1.8–400 K. Therefore, one can postulate that the two hexaferrites are ferrimagnets at room temperature.

**Table 1:** Magnetic Parameters of the SFO and SFN3O Hexaferrites, As Obtained at Room Temperature

composition	saturated magnetization *M*_s_(emu/mg)	remnant magnetization *M*_r_(emu/mg)	coercive field *H*_c_ (Oe)	squareness ratio *M*_rs_
SFO	0.068	0.033	4200	0.485
SFN3O	0.076	0.034	2850	0.447

Figure 4c displays the *M*–*H* loops of the SFO and SFN3O samples measured at room temperature. Both SFO and SFN3O show typical ferrimagnetic behavior. The saturated magnetization *M*_s_ for SFO and SFN3O is 0.068 and 0.076 emu/mg, respectively. Both *M*_s_ and *M*_r_ for SFO are higher than the previous work^9^ possibly due to different preparation methods but agree well with other published work.^15^ According to earlier studies on the SFO-derived hexaferrites,^60^ a high value of *M*_s_ can be ascribed to the high concentration of Fe^3+^ ions in a high spin state and enhanced ferromagnetic exchange interactions between Fe ions caused by decreased oxygen deficiencies.^58^ In this case, nonmagnetic Nb^5+^ ions replacing the Fe ions at 4f_1_ and 4f_2_ sites (spin-down states) give rise to the increased net magnetic moment together due to the enhanced ferromagnetic exchange coupling along the *z*-axis via Fe^3+^–O–Nb^5+^ bonds. This is consistent with fitted XRD results and agrees well with previous findings that Nb or other diamagnetic ions prefer to enter the octahedral and tetrahedral sites of Fe in hexaferrites,^23,61−63^ and the intensity of antiferromagnetic exchange interactions is weakened as the oxygen vacancy decreases.^64,65^

It is expected that both SFO and SFN3O possess multidomain structures with the squareness ratio (*M*_rs_ = *M*_r_/*M*_s_) 0.485 and 0.447, respectively.^61,66^ A slimmer *M*–*H* loop of SFN3O is observed in Figure 4c compared with SFO. According to the domain wall theory,^67^, where *A* is the exchange stiffness, *H*_A_ is the magneto-crystalline anisotropy, *M*_s_ is the saturation magnetization, and *D* is the grain size.^68^ The coercive field *H*_c_ is proportional to the inverse *M*_s_ and smaller grain size.^69,70^ Therefore, one would expect that the dominant reason for a large drop in *H*_c_, nearly 30% of SFN3O, against the initial coercive field of SFO is the increment of saturation magnetization. The room-temperature magnetic parameters of the SFO and SFN3O samples are summarized in Table 1.

**Figure 5:**
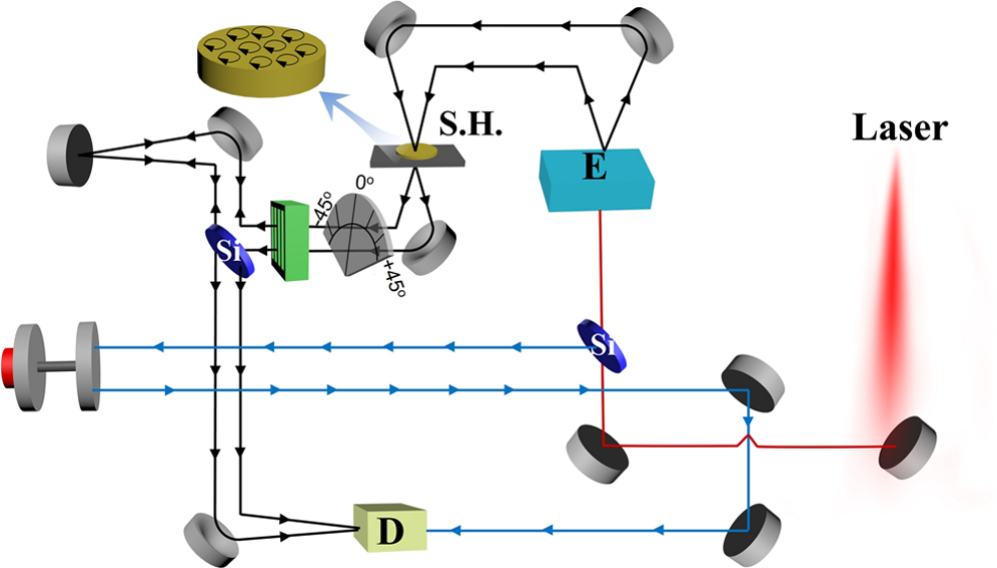
Schematic of the Faraday rotation measurement setup based on the modified THz time-domain spectroscopy. M1–M5: flat reflection mirrors; Si: silicon wafer; E: THz photoconductive emitter; GPM1-GPM5: parabolic mirrors to focus and collimate THz beams; S.H: sample holder; D: THz photoconductive detector; and AM: adjustable retroreflection mirror. The black lines guided by arrows are the transmission THz beam, and the blue lines guided by arrows are the 780 nm probe beam.

**Figure 6:**
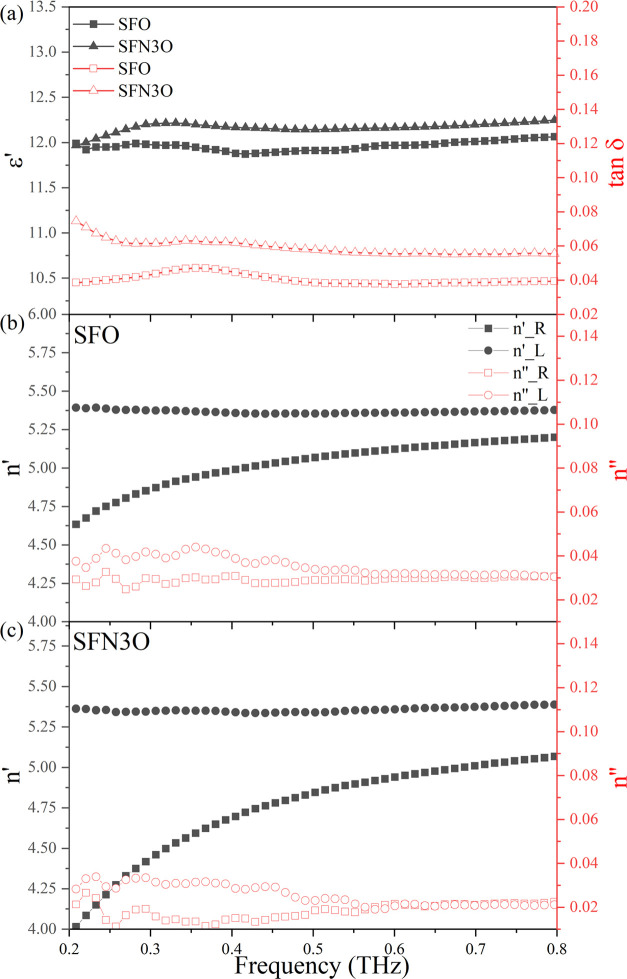
(a) Relative dielectric permittivity and loss tangent of the as-prepared SFO and SFN3O samples at the 0.2–0.8 THz band. (b, c) Complex refractive index of the right- and left-handed directions for the magnetized SFO and SFN3O samples, respectively, at frequencies from 0.2 to 0.8 THz.

A schematic of the setup for measurement of the THz transmission response is shown in Figure 5. Using this setup, the Faraday rotation was determined by the refractive index measured for the left- and right-handed directions after magnetizing the samples at a DC field of 3500 Oe. The permittivity was obtained from the measured refractive index *n* using the following equation
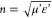
2where μ*′* and ε*′* are the relative permeability and relative permittivity, respectively. Figure 6a shows the frequency dependencies of the dielectric permittivity and loss tangent of the as-prepared (nonmagnetized) SFO and SFN3O samples in the THz band. The permittivity of both hexaferrites is nearly independent of the frequency due to a large ionic polarization and partly because of electronic polarization^71^ within the 0.2–0.8 THz range. A slightly higher value of ε′ and tan δ of SFN3O can be attributed to the smaller grain size effect, reduced coercive field, and higher concentration of the ferrimagnetic active regions at THz frequencies. Figure 6b,c shows the respective complex refractive index for the right- and left-handed directions in the 0.2–0.8 THz range; *n′* is the real part while *n″* is the imaginary part of the refractive index. The value of Δ*n′* (*n′*_left-handed_ – *n′*_right-handed_) is 0.75 for SFO, and it greatly increases to 1.30 for SFN3O. This result clearly demonstrates that the Nb substitution improves the magnetic properties of the pure SFO hexaferrite. Thus, the enhanced magneto-optical behavior, namely the Faraday rotation effect in SFN3O, can be attributed to the higher *M*_s_ and lower *E*_c_. Moreover, the imaginary part of the right-handed refractive index (*n’’*) of SFN3O shows a steeper decline with decreasing frequency than that of SFO, reaching a value of *n’’* of about 0.01 within 0.5 THz. This behavior can be explained by the reduced oxygen vacancies and partial oxidation of Fe^2+^ (Fe^2+^ → Fe^3+^) during thermal treatment.^29^

It can be concluded that the introduction of Nb into M-type hexaferrites is an effective way to improve their room-temperature ferrimagnetic properties and, at the same time, enhance their dielectric behavior in the THz band. The proposed chemical design with donor Nb^5+^ doping in the hexaferrites enables the development of advanced functional materials with improved magneto-optical properties and low dissipation for high-performance imaging and sensing applications in the THz band.

## Conclusions

The M-type hexaferrites of SrFe_12–*x*_Nb_*x*_O_19_ (*x* = 0.00 and 0.03) were prepared by the solid-state reaction. The room-temperature XRD data demonstrate that the two ceramics are single-phase materials with the *P*6_3_/*mmc* space group. The mixed valence states of Fe (Fe^3+^/Fe^2+^) together with oxygen vacancies were revealed by XPS analysis, with SFN3O having fewer oxygen vacancies than SFO. SQUID measurements of the field dependence of magnetization and ZFC/FC magnetization over the temperature range 1.8–400 K evidenced the ferrimagnetic behavior of these two compositions. The increased saturated magnetization (0.076 emu/mg) in the *x* = 0.03 sample was explained by the preferred arrangement of Fe^3+^ ions in the spin-up state. The composition-driven enhancement of both the multidomain structure and ferromagnetic exchange coupling led to a higher saturation and lower coercivity of the Nb-doped hexaferrite. Moreover, this enhanced magnetic performance is accompanied by a large Faraday rotation (Δ*n′* = 1.30) and high relative permittivity in the THz band. Overall, the Nb-doped SrFe_12_O_19_ hexaferrite with excellent magnetic properties in the THz band provides a competitive performance for microwave devices, filters, and recording media.

## Supporting Information Available

Fitted XRD patterns at room temperature; crystal and refinement parameters; fitted results for O 1s and Fe 2p XPS spectra; and DSC thermograms in heating and cooling regimes for SFO and SFN3O (PDF)
