# Supplementary material for: Terahertz Faraday Rotation of SrFe12O19 Hexaferrites Enhanced by Nb Doping
Source: ACS Appl Mater Interfaces. 2022 Oct 4;14(41):46738–47. doi: 10.1021/acsami.2c13088 (PMC9585520; doi:10.1021/acsami.2c13088)
Supplement: Supplementary file 1 — am2c13088_si_001.pdf [file am2c13088_si_001.pdf]

Supporting information

Terahertz Faraday rotation of $SrFe_{12}O_{19}$ hexaferrites enhanced by Nb-doping

Zimeng Hu[a], Gavin B.G. Stenning[b], Vladimir Koval[c], Jiyue Wu[a], Bin Yang[d], Alisa Leavesley[e,f], Richard Wylde[f], Michael John Reece[a], Chenglong Jia[g*], and Haixue Yan[a*]*

[a] School of Engineering and Materials Science, Queen Mary University of London, Mile End Road, London E1 4NS, United Kingdom

[b] ISIS Neutron and Muon Source, Rutherford Appleton Laboratory, Didcot, Oxfordshire OX11 0QX, United Kingdom

[c] Institute of Materials Research, Slovak Academy of Sciences, Kosice 04001, Slovakia

[d] Faculty of Science and Engineering, University of Chester, Parkgate Road, Chester, CH1 4BJ, United Kingdom

[e] Virginia Diodes Inc., 979 2nd St SE #309, Charlottesville, VA 22902, USA

[f] Thomas Keating Ltd, Billingshurst, West Sussex RH14 9SH, United Kingdom

[g] Key Laboratory for Magnetism and Magnetic Materials of MOE, Lanzhou University, Lanzhou730000, P. R. China

*Bin Yang: b.yang@chester.ac.uk

*Chenglong Jia: cljia@lzu.edu.cn

*Haixue Yan: h.x.yan@qmul.ac.uk

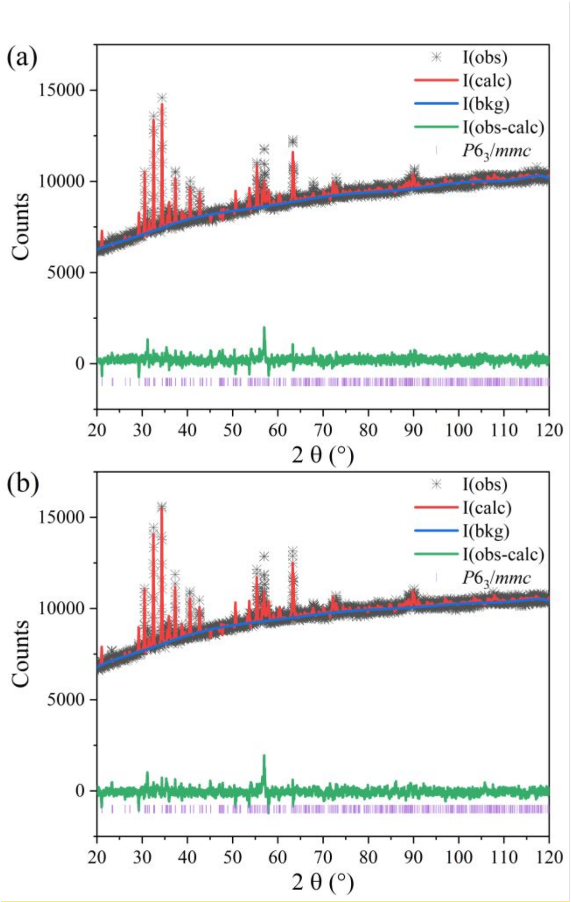

Figure S1. X-ray Rietveld fitted spectra for the SFO (a) and SFN3O (b) at room temperature. Fitted and difference data are plotted in red and green curves, respectively. Vertical lines are Bragg's positions for the hexagonal phase with $P6_3/mmc$ space group.

Table S1. The crystal and refinement parameters for the M-type hexaferrites.

Sample	$SrFe_{12}O_{19}$	$SrFe_{11.97}Nb_{0.03}O_{19}$
Crystal system	Hexagonal	Hexagonal
(Space group)	$P6_3/mmc$	$P6_3/mmc$
Lattice parameters (Å)	a = 5.8884(6)<br>c = 23.0742(3)	a = 5.8870(2)<br>c = 23.0647(4)
Volume (Å^3)	692.887(2)	692.262(3)
R – factors	R_{wp} = 0.0146<br>R_p = 0.0105<br>R_{exp} = 0.009	R_{wp} = 0.0143<br>R_p = 0.0101<br>R_{exp}= 0.009
χ^2	2.665	2.682
No. of profile points	3649	3649

Table S2. The fitting results of O 1s XPS spectra for the M-type hexaferrites.

Composition		O_{latt}	O_{vac}	O_{abs}	O_{vac}:O_{latt}
SFO	B.E. (eV)	529.40	530.82	532.78	0.354(3)
	Area (%)	71.80	25.44	2.76	
SFN3O	B.E. (eV)	529.56	530.86	532.99	0.222(1)
	Area (%)	79.67	17.70	2.63	

Note: B.E. is binding energy.

Table S3. The fitting results of Fe 2p XPS spectra for the M-type hexaferrites.

Composition		Fe^{2+} $2p_{3/2}$	Fe^{3+} $2p_{3/2}$	Fe^{2+} $2p_{1/2}$	Fe^{3+} $2p_{1/2}$
SFO	B.E. (eV)	709.18	710.88	720.18	723.48
	Area (%)	51.85	48.15	38.46	61.54
SFN3O	B.E. (eV)	709.38	711.08	720.38	723.78
	Area (%)	37.20	62.80	36.10	63.90

Note: B.E. is binding energy.

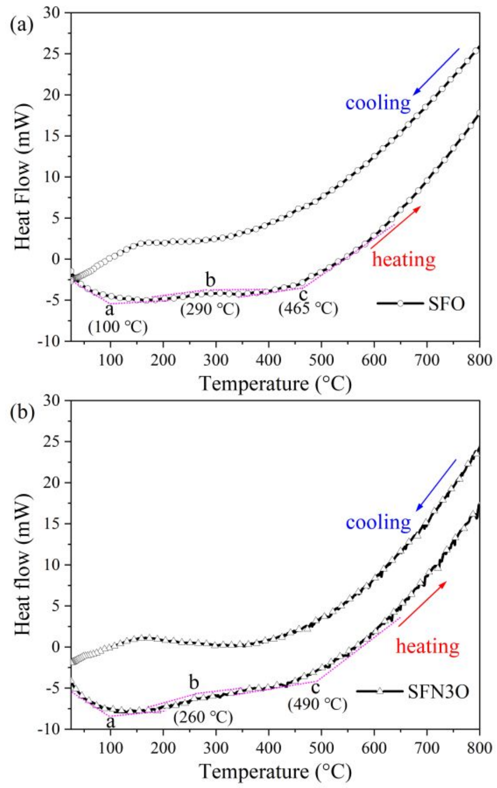

Figure S2. DSC thermograms (in heating and cooling regimes) for SFO (a) and SFN3O (b), respectively.
